# Age-Based Left-Digit Bias in the Treatment of Pancreatic Adenocarcinoma

**DOI:** 10.1007/s12029-026-01459-1

**Published:** 2026-04-13

**Authors:** Qianyun Luo, Bethel Ozed-Williams, David G. Brauer, Bryan Trottier, Todd M. Tuttle, Jane Y. C. Hui, Eric H. Jensen, Jacob S. Ankeny, Christopher J. LaRocca, Schelomo Marmor

**Affiliations:** 1 Division of Surgical Oncology, University of Minnesota, 420 Delaware St. SE, MMC 195, Minneapolis, MN 55455 USA; 2 University of Minnesota Medical School, 420 Delaware Street SE, Minneapolis, Minnesota 55455 United States of America

**Keywords:** Decision Making, Health Disparities, Geriatric Assessment, Cognitive Bias, Chemotherapy

## Abstract

**Purpose:**

Left digit bias, where the left-most digit disproportionately influences decision-making, can impact treatment decisions and patient outcomes. This study examines how such bias may affect treatment decisions for patients with pancreatic adenocarcinoma (PDAC), focusing on differences between patients aged 79 and 80.

**Method:**

A retrospective cohort study using the National Cancer Database (2004–2020) analyzed patients with PDAC aged 79 and 80. The primary exposure was age-based left-digit bias. The main outcomes measured included differences in treatment received and overall survival. Statistical analyses included chi-square tests, regression discontinuity analysis, multivariate analysis, Kaplan-Meier survival curves, and Cox proportional hazards models.

**Results:**

Among 5,304 patients (2,718 aged 79, 2,586 aged 80), chemotherapy use was higher in 79-year-olds (50.3%) than 80-year-olds (45.1%) (OR 0.79, 95% CI 0.70–0.89, *p* < 0.01). No significant differences were found in surgery or radiation. Chemotherapy was associated with lower mortality risk (HR 0.85, 95% CI 0.79–0.92, *p* < 0.01). Adjusted overall survival was similar between groups (HR 1.00, 95% CI 0.93–1.07, *p* = 0.91).

**Conclusion:**

Left digit bias was observed in the treatment of PDAC, with a tendency to favor chemotherapy for patients aged 79 over 80. This highlights the need for age-neutral decision-making in treatment planning to avoid biases that could impact patient care and outcomes.

**Supplementary Information:**

The online version contains supplementary material available at 10.1007/s12029-026-01459-1.

## Background

Heuristics, mental shortcuts used to simplify decision-making, are pervasive across various domains and often lead to cognitive biases [1–3]. It was once believed that highly trained professionals, such as physicians, would be less susceptible to these biases due to their expertise [4–7]. However, emerging research indicates that physicians, despite their advanced training, are also influenced by heuristics, especially under conditions of time pressure, uncertainty, and cognitive fatigue [8–10]. This underscores the need to better understand how these biases impact clinical decision-making and patient outcomes.

One specific heuristic identified in medical settings is left-digit bias. This bias occurs when decisions are disproportionately influenced by the left-most digit of numerical values, such as age or blood pressure [8–12]. For example, a price of $1.99 is often perceived as significantly lower than $2.00, despite the minimal difference [13]. Although extensively studied in economics, the impact of left-digit bias in medical decision-making is less understood. Recent studies suggest that physicians may be more likely to recommend certain treatments, such as coronary artery bypass graft surgery, for patients aged 79 compared to those aged 80, even when their clinical conditions are similar [8, 11]. This effect has also been observed in other medical decisions, including emergency department protocols, kidney transplantation, and surgery for acute cholecystitis [8, 12, 13].

Age-based bias significantly impacts the management and outcomes of pancreatic ductal adenocarcinoma (PDAC) which often presents at an advanced stage with a 5-year mortality rate of about 90% [14–19]. Current therapies involve a combination of surgical resection of PDAC with adjuvant chemoradiation for optimal outcomes. However, less than 25% of patients are considered resectable upon diagnosis [14, 15, 17], a proportion that declines with advancing age [17–19]. Moreover, only about 35% of individuals aged 70 and older with resectable disease undergo potentially curative surgery [17, 20, 21]. and fewer than 50% of patients aged 80 or older receive adjuvant chemotherapy post-resection [17, 18, 21].

Recognizing that age bias and deviations from established guidelines can exacerbate disparities in cancer treatment [18, 19, 22], our study aims to investigate the influence of left-digit bias on treatment decisions for older patients with PDAC. Prior studies have shown that individuals over 80 years old have comparable survival outcomes to younger patients when treatment, specifically, adjuvant chemotherapy is administered [17, 20, 23]. Some studies have identified frailty as a crucial determinant influencing treatment decisions and outcomes in PDAC [14, 15, 17]. Nonetheless, age has emerged as an independent predictor of poorer outcomes, even after controlling for sociodemographic and clinical confounders [17]. Thus it is apparent that treatment choices often vary based on patient characteristics and physician judgment but the extent to which heuristic age bias contributes to disparities in guideline-concordant PDAC care remains inadequately explored.

Our primary objective was to assess if treatment approaches differed between patients aged 79 and those aged 80. This study is the first to investigate heuristic age-based bias specifically within the context of PDAC, a highly aggressive malignancy with generally poor prognosis. We hypothesize that in patients with minimal medical comorbidities, treatment decisions for patients aged 80 will not significantly differ from those made for patients aged 79.

## Methods

### Data and Population

We used the National Cancer Database (NCDB) to select patients with primary PDAC between 2004 and 2020. The NCDB is a joint project of the Commission on Cancer (CoC) of the American College of Surgeons and the American Cancer Society. The CoC’s NCDB and the hospital participating in the CoC NCDB are the source of the deidentified data used herein; they have not verified and are not responsible for the statistical validity of the data analysis or the conclusions derived by the authors. The NCDB contains over 30 million records of individual cancer cases collected by more than 1500 CoC-approved facilities across the United States. The NCDB is estimated to capture approximately 70% of all newly diagnosed cases of cancer in the United States. The University of Minnesota’s institutional review board has deemed analysis of the NCDB dataset exempt from review.

Patients with stage I-II PDAC were included in the study to focus on potentially resectable tumors and ensure a more homogeneous study population, thus reducing confounding factors related to advanced disease stages. Patients with metastatic disease were excluded. The study population was categorized into two cohorts based on age: patients aged 79 years and patients aged 80 years. This categorization was chosen to investigate potential left-digit bias in treatment patterns.

To further validate findings, a sensitivity analysis was conducted on individuals with a Charlson-Deyo comorbidity score of 1 or less, ensuring robustness in the assessment of treatment differences. The NCDB version of the Charlson-Deyo Score excludes cancer diagnoses from scoring. Thus, a Charlson-Deyo score of 0 or 1 in this dataset reflects minimal non-cancer-related comorbidities.

### Statistical Analysis

Cohorts were compared using chi-square tests for categorical variables, including demographic factors (sex, race/ethnicity, primary payer, and Charlson-Deyo comorbidity score), disease-specific factors (tumor grade), and treatment modalities (surgical management, chemotherapy, and radiation therapy). The receipt of treatment modalities was considered at any stage of the patient’s disease course. Additionally, we examined the documented reasons for which chemotherapy was not chosen in each age group. Documented chemotherapy refusal was analyzed descriptively to provide contextual information. Chemotherapy refusal was not included in multivariable models because it is only recorded among patients who did not receive chemotherapy in the NCDB and is therefore structurally colinear with the outcome. Multivariable logistic regression was employed to evaluate variables associated with treatment patterns.

Left-digit bias was examined using regression discontinuity (RD) analyses with age as the running variable and a cutoff at 80 years, comparing patients immediately below versus above the decade boundary. Both parametric local linear and nonparametric bias-corrected regression discontinuity estimators were used to evaluate the robustness of the discontinuity given the discrete nature of the age variable.

OS stratified by age and chemotherapy use was evaluated with Kaplan–Meier and Cox proportional hazards modeling and to assess the impact of left-digit bias on survival outcomes among the two age groups. To mitigate selection bias of patients too frail to receive treatment, patients with three months of survival or less were excluded from the survival analysis. Analyses were performed with R, Version 4.3.0 (R Foundation for Statistical Computing; Vienna, Austria). A two tailed p-value of ≤ 0.05 was selected to reflect statistical significance.

## Results

### Receipt of Treatment

We identified 5,304 PDAC patients with 2,718 patients aged 79 and 2,586 patients aged 80. Eighty-year-old patients were significantly less likely to receive treatment compared to 79-year-olds (33% vs. 36%, *p* = 0.01). The impact of age on treatment was more pronounced for chemotherapy. Patients aged 80 years were less likely to receive chemotherapy compared to 79-year-olds (45% vs. 50%, *p* < 0.01, Table 1). Chemotherapy was defined as receipt of systemic therapy at any point in the treatment course. When disaggregated by treatment sequence, 14% of 79-year-olds received adjuvant chemotherapy alone, 4% received neoadjuvant chemotherapy alone, and 2% received both. Corresponding rates among 80-year-olds were 12%, 3%, and 2%, respectively (*p* = 0.07). Chemotherapy utilization was lower among 80-year-olds across all systemic treatment sequences. However, there was no difference in the receipt of surgery or radiation therapy between the two age groups (Table 1).

**Table 1:** Patient demographics for patients with PDAC in the NCDB from 2004–2020

		age 79 (*n* = 2,718)		age 80 (*n* = 2,586)		
		*n*	%	*n*	%	Crude OR (95% CI)
Sex	Male	1188	43	1131	44	1 (0.90–1.12)
	Female	1530	56	1455	56	REF
Race/Ethnicity	White Non-Hispanic	2138	79	2040	79	REF
	Black Non-Hispanic	235	9	226	9	1.01 (0.83–1.22)
	Asian/ Pacific Islander/ American Indian	74	3	72	3	1.02 (0.73–1.42)
	Other	271	10	248	10	0.96 (0.80–1.15)
Primary Payor	Private	237	9	218	8	REF
	Medicare	2338	86	2257	87	1.05 (0.87–1.27)
	Medicaid / Other Government	74	3	48	2	0.71 (0.47–1.06)
	Not Insured/Unknown	69	3	63	3	0.99 (0.67–1.46)
Grade	Well-differentiated	122	4	107	4	REF
	Moderately differentiated	522	19	494	19	1.08 (0.81–1.44)
	Poorly differentiated and undifferentiated	407	15	326	13	0.91 (0.68–1.23)
	Other/Unknown	1667	61	1659	64	1.13 (0.87–1.48)
Receipt of Chemotherapy	Yes	1368	50	1167	45	0.82 (0.74–0.92)
	No	1242	46	1290	50	REF
	Unknown	108	4	129	5	1.15 (0.88–1.50)
Surgery	None	1836	68	1806	70	REF
	Whipple	558	21	490	19	0.89 (0.78–1.02)
	Partial pancreatectomy	131	5	131	5	1.02 (0.79–1.31)
	Other/Unknown	193	7	159	6	0.84 (0.67–1.04)
Radiation therapy	No	2137	79	2018	78	REF
	Yes	506	19	481	19	1.02 (0.88–1.16)
	Unknown	74	3	87	3	1.25 (0.91–1.71)
Any treatment	No	887	33	930	36	REF
	Yes	1831	67	1656	64	0.86 (0.77–0.96)
Charlson-Deyo Score	0	1708	63	1581	61	REF
	1	640	24	650	25	1.10 (0.96–1.25)
	2+	370	14	355	14	1.04 (0.88–1.22)

Among 79-year-olds, 32% received chemotherapy with or without radiation (no surgery), while this decreased to 30% for 80-year-olds. Notably, the proportion of patients receiving no treatment increased from 37% in 79-year-olds to 41% in 80-year-olds. Radiation alone was administered to 2% of 79-year-olds and 3% of 80-year-olds. Surgery with or without adjuvant therapy was performed in 29% of 79-year-olds, compared to 26% of 80-year-olds. To evaluate whether the observed difference between 79- and 80-year-olds reflects a discontinuity suggestive of left-digit bias rather than a gradual age-related decline, we compared treatment rates between adjacent age groups. Chemotherapy receipt was similar between 78- and 79-year-olds (30% vs. 32%), and between 80- and 81-year-olds (30% vs. 29%), with no statistically significant differences. The proportion of patients receiving no treatment increased gradually across these age groups (35% at 78, 37% at 79, 41% at 80, and 43% at 81), but the largest jump occurred specifically between 79 and 80. These findings suggest a potential inflection point at age 80, consistent with a left-digit bias.

When chemotherapy utilization was examined across broader age decades (50s, 60s, 70s, 80s, and 90s), a progressive decline in chemotherapy receipt was observed with advancing age (Supplementary Table S1). The largest absolute reduction occurred between patients in their 70s and 80s, corresponding to the transition into octogenarian status (Table S1).

**Fig. 1:**
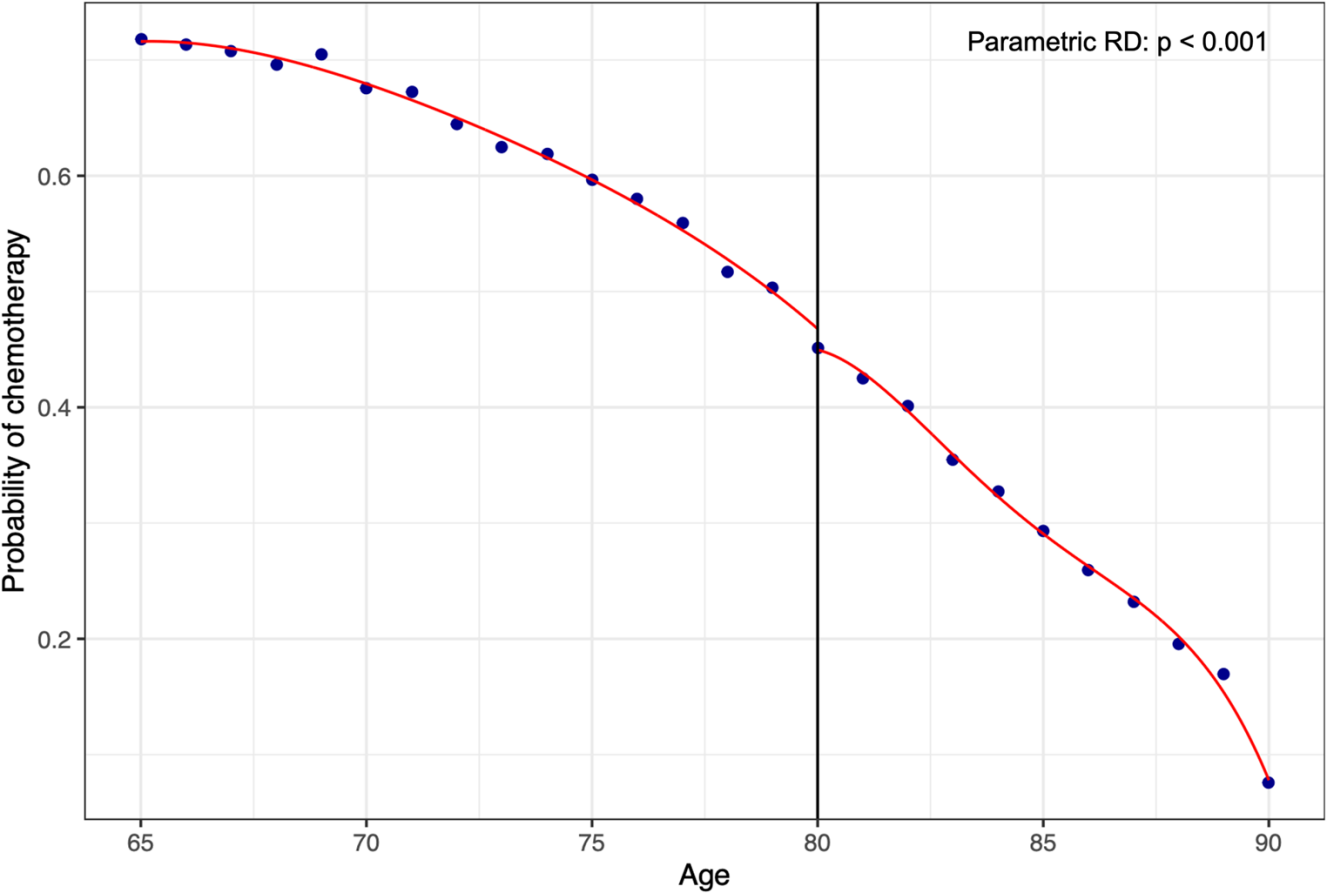
Regression discontinuity analysis of chemotherapy receipt at age 80 among patients with stage I–II pancreatic ductal adenocarcinoma. Points represent observed age-specific proportions; solid lines represent parametric local linear fits on either side of the 80-year cutoff (*p* < 0.001)

To formally evaluate the presence of a threshold effect, we conducted RD analyses treating age as a continuous running variable centered at 80 years. Crossing the age-80 threshold was associated with an absolute 4.7-percentage-point reduction in chemotherapy receipt. This estimate was statistically significant in parametric local linear RD models (*p* < 0.001, Fig. 1). However, nonparametric RD with bias-corrected inference produced a directionally consistent but not statistically significant estimate (robust *p* = 0.09). No statistically significant discontinuities were observed at the 59/60 or 69/70 decade boundaries, and the 89/90 boundary could not be evaluated due to aggregation of patients aged ≥ 90 years in the NCDB.

Multivariate analysis, adjusting for covariates including sex, race/ethnicity, primary payer, Charlson-Deyo comorbidity score, tumor grade, surgical management, and radiation therapy, indicates significant differences in the receipt of chemotherapy with 45% of patients aged 80 and 50% of patients aged 79 receiving chemotherapy (aOR 0.79, 95% CI 0.70–0.89, *p* < 0.01, Table 2). A sensitivity analysis restricted to patients with a Charlson-Deyo comorbidity score of 1 or less yielded consistent results (aOR, 0.77; 95% CI, 0.68–0.88; *p* < 0.01). However, there was no difference in the receipt of surgery or radiation therapy between 79- and 80-year-olds (Table 2). Although we could not fully account for refusal of chemotherapy and clinical decision making, we observed that 14% of patients in the 79-year-old group refused chemotherapy while 18% of 80-year-old refused chemotherapy (*p* < 0.01).

**Table 2:** Multivariable logistic regression analysis of treatment receipt among patients with stage I–II PDAC in the NCDB, 2004–2020.The analysis compares odds of receiving treatment across demographic and clinical covariates. Age 79 was used as the reference group for age. Variables with statistically significant associations (*p* < 0.05) are highlighted

Chemotherapy		aOR age 80/79	95% CI	*p*-value	
	No	REF			
	Yes	0.79	0.70–0.89	**< 0.01**	
Sex	Male	1.01	0.90–1.12	0.91	
	Female	REF			
Race/Ethnicity	White Non-Hispanic	REF			
	Black Non-Hispanic	0.99	0.81–1.2	0.88	
	Asian / Pacific Islander / American Indian	1.05	0.75–1.46	0.78	
	Other	0.96	0.79–1.15	0.63	
Primary Payor	Private	REF			
	Medicare	1.05	0.87–1.28	0.62	
	Medicaid / Other Government	0.7	0.47–1.06	0.09	
	Not Insured/Unkown	0.99	0.67–1.46	0.95	
Grade	Well-differentiated	REF			
	Moderately differentiated	1.08	0.81–1.45	0.6	
	Poorly differentiated and undifferentiated	0.92	0.68–1.24	0.57	
	Other/Unknown	1.11	0.84–1.47	0.47	
Surgery	None	REF			
	Whipple	0.99	0.83–1.19	0.95	
	Partial pancreatectomy	1.11	0.84–1.48	0.46	
	Other	0.88	0.69–1.13	0.31	
	unknown	0.76	0.40–1.48	0.42	
Radiation therapy	No	REF			
	Yes	1.12	0.96–1.30	0.14	
	Unknown	1.25	0.91–1.73	0.17	
Charlson-Deyo Score	0	REF			
	1	1.09	0.96–1.25	0.17	
	2+	1.02	0.86–1.20	0.83	

## Overall Survival

**Fig. 2:**
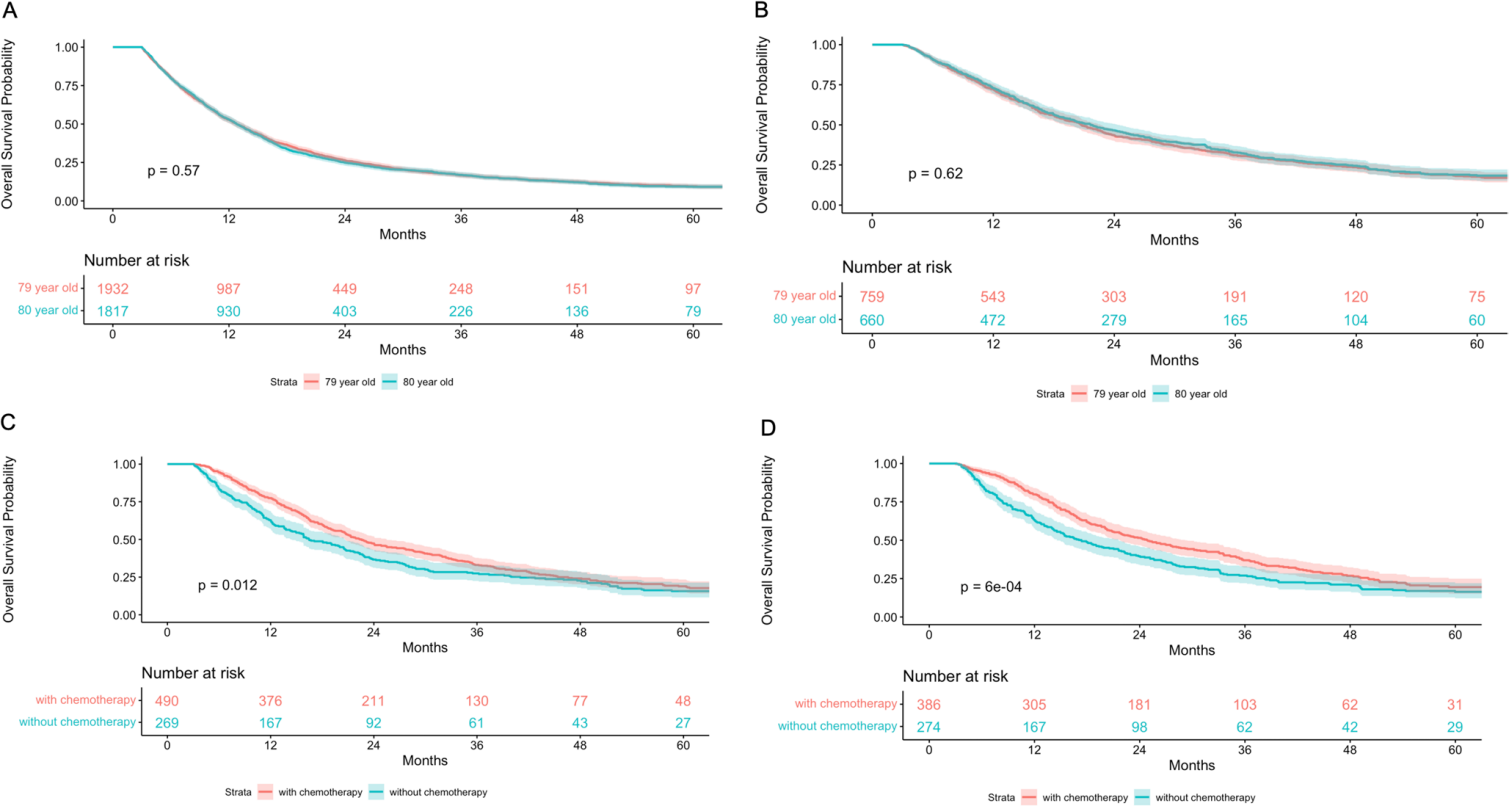
Kaplan Meier Survival Curve based on age and chemotherapy (**A**: Comparison of survival between 79-year-old and 80-year-old patients. **B**: Survival comparison between 79-year-old and 80-year-old patients who underwent surgery. **C**: Survival of 79-year-old patients who underwent surgery, comparing those who received chemotherapy vs. those who did not. **D**: Survival of 80-year-old patients who underwent surgery, comparing those who received chemotherapy vs. those who did not.)

The median overall survival (OS) showed no significant difference between 79-year-olds (12.3 months) and 80-year-olds (12.4 months) in the full cohort (*p* = 0.57, Fig. 2A). Among patients who underwent surgical resection, median OS was comparable between 79-year-olds (19.1 months) and 80-year-olds (20.1 months), with no statistically significant difference (*p* = 0.62, Fig. 2B). In the surgically resected subgroup, 79-year-old patients who received adjuvant chemotherapy achieved a median OS of 20.8 months, compared to 16.1 months for those who did not receive chemotherapy (*p* = 0.01, Fig. 2C). A similar pattern was observed in 80-year-old surgical patients, with median OS of 22.0 months and 15.7 months for those with and without chemotherapy, respectively (*p* < 0.01, Fig. 2D). Cox proportional hazards analysis revealed that chemotherapy administration was associated with a significantly reduced mortality risk (HR 0.84, 95% CI: 0.79–0.91, *p* < 0.01, Table 3). After controlling for treatment modality, no significant survival difference was detected between the age groups (Fig. 2A and B). In the adjusted Cox model accounting for treatment and demographic factors, overall survival remained comparable between 79- and 80-year-olds (HR 0.99, 95% CI: 0.93–1.07, *p* = 0.86, Table 3).

**Table 3:** Cox proportional hazards model of overall survival among patients with stage I–II PDAC in the NCDB, 2004–2020. Hazard ratios were estimated to assess the association of clinical and demographic characteristics with overall survival

Chemotherapy		Hazard ratio	95% CI	*p*-value
	No	REF		
	Yes	0.84	0.79–0.91	< 0.01
Age	79	REF		
	80	0.99	0.93–1.07	0.86
Sex	Male	1.04	0.97–1.12	0.26
	Female	REF		
Race/Ethnicity	White Non-Hispanic	REF		
	Black Non-Hispanic	0.9	0.80–1.02	0.11
	Asian / Pacific Islander / American Indian	0.74	0.59–0.91	0.01
	Other	0.9	0.79–1.01	0.08
Primary Payor	Private	REF		
	Medicare	1.11	0.98–1.26	0.1
	Medicaid / Other Government	0.87	0.66–1.17	0.36
	Not Insured/Unknown	1	0.78–1.27	0.97
Grade	Well-differentiated	REF		
	Moderately differentiated	1.18	0.98–1.41	0.08
	Poorly differentiated and undifferentiated	1.39	1.14–1.67	< 0.01
	Other/Unknown	1.09	0.91–1.30	0.35
Surgery	None	1	REF	REF
	Whipple	0.38	0.34–0.42	< 0.01
	Partial pancreatectomy	0.35	0.29–0.41	< 0.01
	Other	0.41	0.35–0.48	< 0.01
Radiation therapy	No	REF		
	Yes	0.89	0.81–0.97	0.01
	Unknown	1	0.83–1.21	0.99
Charlson-Deyo Score	0	REF		
	1	1.12	1.03–1.22	0.01
	2+	1.23	1.11–1.38	< 0.01

## Discussion

In this national cohort of patients with stage I-II PDAC, we identified a discrete difference in chemotherapy receipt between patients aged 79 and 80 years. Although chemotherapy use declined progressively across advancing decades of life, the most pronounced reduction occurred at the transition into octogenarian status. Importantly, no differences were observed in surgical utilization, radiation therapy, or adjusted overall survival between these adjacent age groups.

Age based bias contributes to significant disparities in patient care [18–20, 22, 24]. While physicians are highly trained, research shows that they can still be vulnerable to cognitive biases, particularly when facing uncertainty with complex pathologies like PDAC. One such bias is left-digit bias, where decisions are disproportionately influenced by the left-most digit in a numerical value, such as a patient’s age. In this study, we explored the potential left digit bias in the treatment of 79-year-olds vs. 80-year-olds with PDAC. Although we observed no difference in the utilization of surgery or radiation therapy—suggesting that these patients were considered medically appropriate for intensive treatment—50% of 79-year-olds received chemotherapy compared with 45% of 80-year-olds, a statistically significant difference indicating that the left-most digit in age may disproportionately influence treatment decisions for older patients. After adjusting for relevant demographic and clinical factors, this bias persisted, with patients aged 79 being more likely to receive chemotherapy (adjusted odds ratio 0.79, 95% CI 0.70–0.89).

Across broader age decades, chemotherapy utilization declined progressively from the 50s through the 90s, with the largest absolute reduction observed between patients in their 70s and 80s (Supplementary Table S1). Age 80 represents a widely recognized clinical milestone and approximates U.S. life expectancy [25], which may function as a salient cognitive threshold in clinical decision-making beyond gradual biological aging alone. Formal regression discontinuity analyses were conducted at multiple decade thresholds (59/60, 69/70, and 79/80). No significant discontinuities were observed at 59/60 or 69/70. At the 79/80 boundary, crossing age 80 was associated with a 4.7-percentage-point reduction in chemotherapy receipt. This estimate was statistically significant in parametric models but attenuated under nonparametric bias-corrected estimation, reflecting the modest magnitude of the effect and the discrete nature of the age variable.

Notably, we observed no difference in surgical or radiation therapy utilization between patients aged 79 and 80 years. Given that surgical resection carries the highest risk of perioperative morbidity and mortality among PDAC treatment modalities, the absence of surgical differences suggests that treatment variation at age 80 is modality-specific rather than reflective of a generalized aversion to aggressive care. Chemotherapy decisions, which require individualized assessments of tolerance and anticipated benefit, may be more susceptible to subtle age-based heuristics. Similarly, radiation therapy utilization did not differ between the two age groups, further supporting that the observed treatment variation at age 80 was specific to chemotherapy rather than reflective of a generalized reduction in aggressive care.

Given that cognitive heuristics have been shown to influence physician decision-making across clinical contexts [1–3, 8–13], structured clinical decision-support tools may help reduce unintended reliance on categorical age thresholds while preserving individualized clinical judgment. User centered design methodology as part of decision support tools has been implemented in other areas of health services and health care technology [26–31], and approaches may help ensure that treatment decisions are guided by objective clinical factors rather than salient numerical boundaries.

This study has several limitations inherent to retrospective analyses of the NCDB. The database lacks granular clinical variables such as ECOG performance status, frailty indices, cognitive function, social support, and postoperative complication data, all of which may influence chemotherapy eligibility and decision-making [14, 15, 17, 32]. Several studies have shown that up to 50% of patients with pancreatic cancer do not receive adjuvant chemotherapy, often due to postoperative morbidity or patient non-compliance [33]. These findings underscore the importance of postoperative recovery and functional status in determining adjuvant therapy receipt. Because the NCDB does not capture detailed postoperative complication severity or recovery trajectories, we cannot exclude these mechanisms as contributors to reduced chemotherapy utilization among 80-year-old patients. Although we adjusted for Charlson-Deyo comorbidity score and performed sensitivity analyses restricted to patients with minimal comorbidity, residual confounding from unmeasured physiologic reserve, postoperative recovery, or social support cannot be excluded. Documented chemotherapy refusal was more frequent among 80-year-olds (18% vs. 14%), which may partially contribute to lower chemotherapy utilization; however, refusal data are limited and do not fully account for the observed difference. Additionally, although race/ethnicity was included in the multivariate analysis, the sample size within age-race strata was too small to support further subgroup analysis comparing chemotherapy receipt between racial/ethnic groups. Therefore, we could not assess whether left-digit bias operates similarly across racial groups or contributes to disparities in treatment decisions. Lastly, while chemotherapy regimens for PDAC have evolved during the 2004–2020 study period, the overall distribution of treatment modalities remained stable, supporting inclusion of the full timeframe. Nonetheless, temporal practice variation cannot be entirely excluded. Despite these limitations, our results shed light on the influence of left-digit bias in treatment decisions, highlighting the need for more nuanced and age-neutral approaches in clinical practice.

## Conclusion

We found evidence of a discrete difference in chemotherapy receipt between patients aged 79 and 80 years with pancreatic ductal adenocarcinoma. The observed variation at this one-year threshold is consistent with the possibility of left-digit bias, whereby treatment decisions may be influenced by the left-most digit of age rather than by a smooth progression of clinical risk. These findings highlight how subtle cognitive heuristics may shape complex oncologic decision-making. Increasing clinician awareness of left-digit bias and related cognitive influences may help ensure that treatment recommendations are grounded in individualized clinical assessment rather than categorical age thresholds. In addition, thoughtfully designed clinical decision-support tools may help promote consistent and patient-centered treatment planning, particularly in older adults with high-risk malignancies. 

## Supplementary Information

Below is the link to the electronic supplementary material.

Supplementary Material 1 (DOCX 12.5 KB)

## Data Availability

The data that support the findings of this study are available from the National Cancer Database but restrictions apply to the availability of these data, which were used under license for the current study, and so are not publicly available. Data are however available from the authors upon reasonable request and with permission of the National Cancer Database.

## References

[1] Tversky A, Kahneman D. Judgment under Uncertainty: Heuristics and Biases: Biases in judgments reveal some heuristics of thinking under uncertainty. Science. 1974;185:1124–31. 10.1126/science.185.4157.1124. 17835457 10.1126/science.185.4157.1124

[2] Kahneman D, Tversky A. On the study of statistical intuitions. Cognition. 1982;11:123–41. 10.1016/0010-0277(82)90022-1. 7198957 10.1016/0010-0277(82)90022-1

[3] Gilovich T, Griffin D, Kahneman D, editors. Heuristics and Biases: The Psychology of Intuitive Judgment [Internet]. 1st ed. Cambridge University Press; 2002. [cited 2024 Dec 20]. 10.1017/CBO9780511808098.

[4] List JA. Does Market Experience Eliminate Market Anomalies? Q J Econ. 2003;118:41–71. 10.1162/00335530360535144.

[5] Alevy JE, Landry CE, List JA, FIELD EXPERIMENTS ON, THE ANCHORING OF ECONOMIC VALUATIONS. Econ Inq. 2015;53:1522–38. 10.1111/ecin.12201.

[6] Lacetera N, Pope DG, Sydnor JR. Heuristic Thinking and Limited Attention in the Car Market. The American Economic Review. Am Economic Association. 2012;102:2206–36.

[7] Dror IE. The paradox of human expertise: why experts get it wrong. In: Kapur N, editor. The Paradoxical Brain [Internet]. Cambridge: Cambridge University Press; 2011. pp. 177–88. 10.1017/CBO9780511978098.011.

[8] Dalmacy DM, Diaz A, Hyer M, Pawlik TM. Age-Based Left-Digit Bias in the Management of Acute Cholecystitis. J Gastrointest Surg. 2021;25:3239–41. 10.1007/s11605-021-05065-3. 34173162 10.1007/s11605-021-05065-3

[9] Marewski JN, Gigerenzer G. Heuristic decision making in medicine. Dialog Clin Neurosci. 2012;14:77–89. 10.31887/DCNS.2012.14.1/jmarewski. 10.31887/DCNS.2012.14.1/jmarewski PMC3341653 22577307

[10] Redelmeier DA. Medical Decision Making in Situations That Offer Multiple Alternatives. JAMA. 1995;273:302. 10.1001/jama.1995.03520280048038. 7815657 10.1001/jama.1995.03520280048038

[11] Olenski AR, Zimerman A, Coussens S, Jena AB. Behavioral Heuristics in Coronary-Artery Bypass Graft Surgery. N Engl J Med. 2020;382:778–9. 10.1056/NEJMc1911289. 32074429 10.1056/NEJMc1911289 PMC7312750

[12] Husain SA, King KL, Mohan S. Left-digit bias and deceased donor kidney utilization. Clin Transplant. 2021;35:e14284. 10.1111/ctr.14284. 33705569 10.1111/ctr.14284 PMC9162444

[13] Coussens S. Behaving Discretely: Heuristic Thinking in the Emergency Department. SSRN Journal [Internet]. 2018 [cited 2024 Dec 20]; 10.2139/ssrn.3743423.

[14] Blackford AL, Canto MI, Klein AP, Hruban RH, Goggins M. Recent Trends in the Incidence and Survival of Stage 1A Pancreatic Cancer: A Surveillance, Epidemiology, and End Results Analysis. JNCI: J Natl Cancer Inst. 2020;112:1162–9. 10.1093/jnci/djaa004. 31958122 10.1093/jnci/djaa004 PMC7669234

[15] Stoffel EM, Brand RE, Goggins M. Pancreatic Cancer: Changing Epidemiology and New Approaches to Risk Assessment, Early Detection, and Prevention. Gastroenterology. 2023;164:752–65. 10.1053/j.gastro.2023.02.012. 36804602 10.1053/j.gastro.2023.02.012 PMC10243302

[16] National Cancer Institute. Surveillance, Epidemiology, and End Results Program [Internet]. https://seer.cancer.gov/

[17] Nipp R, Tramontano AC, Kong CY, Pandharipande P, Dowling EC, Schrag D, et al. Disparities in cancer outcomes across age, sex, and race/ethnicity among patients with pancreatic cancer. Cancer Med. 2018;7:525–35. 10.1002/cam4.1277. 29322643 10.1002/cam4.1277 PMC5806100

[18] Malik AK, Lamarca A, Siriwardena AK, O’Reilly D, Deshpande R, Satyadas T, et al. The Influence of Patients’ Age on the Outcome of Treatment for Pancreatic Ductal Adenocarcinoma. Pancreas. 2020;49:201–7. 10.1097/MPA.0000000000001486. 32011535 10.1097/MPA.0000000000001486

[19] Lima HA, Alaimo L, Moazzam Z, Endo Y, Woldesenbet S, Katayama E, et al. Disparities in NCCN Guideline-Compliant Care for Patients with Early-Stage Pancreatic Adenocarcinoma at Minority-Serving versus Non-Minority-Serving Hospitals. Ann Surg Oncol. 2023;30:4363–72. 10.1245/s10434-023-13230-y. 36800128 10.1245/s10434-023-13230-y

[20] Mehtsun WT, McCleary NJ, Maduekwe UN, Wolpin BM, Schrag D, Wang J. Patterns of Adjuvant Chemotherapy Use and Association With Survival in Adults 80 Years and Older With Pancreatic Adenocarcinoma. JAMA Oncol. 2022;8:88. 10.1001/jamaoncol.2021.5407. 34854874 10.1001/jamaoncol.2021.5407 PMC8640950

[21] Weir HK, Thompson TD, Stewart SL, White MC. Cancer Incidence Projections in the United States Between 2015 and 2050. Prev Chronic Dis. 2021;18:210006. 10.5888/pcd18.210006. 10.5888/pcd18.210006 PMC8220959 34114543

[22] Williams CP, Kenzik KM, Azuero A, Williams GR, Pisu M, Halilova KI, et al. Impact of Guideline-Discordant Treatment on Cost and Health Care Utilization in Older Adults with Early-Stage Breast Cancer. Oncologist. 2019;24:31–7. 10.1634/theoncologist.2018-0076. 30120157 10.1634/theoncologist.2018-0076 PMC6324646

[23] Smith BD, Smith GL, Hurria A, Hortobagyi GN, Buchholz TA. Future of Cancer Incidence in the United States: Burdens Upon an Aging, Changing Nation. JCO. 2009;27:2758–65. 10.1200/JCO.2008.20.8983. 10.1200/JCO.2008.20.8983 19403886

[24] Marmor S, Burke EE, Virnig BA, Jensen EH, Tuttle TM. A comparative analysis of survival outcomes between pancreatectomy and chemotherapy for elderly patients with adenocarcinoma of the pancreas. Cancer. 2016;122:3378–85. 10.1002/cncr.30199. 27419382 10.1002/cncr.30199

[25] Schneider L. JAMA. 2025;334:849. 10.1001/jama.2025.10994. US Life Expectancy Is Rebounding. 10.1001/jama.2025.10994 40779261

[26] Finlayson E, Fan Z, Birkmeyer JD. Outcomes in Octogenarians Undergoing High-Risk Cancer Operation: A National Study. J Am Coll Surg. 2007;205:729–34. 10.1016/j.jamcollsurg.2007.06.307. 18035254 10.1016/j.jamcollsurg.2007.06.307

[27] Graham AK, Lattie EG, Mohr DC. Experimental Therapeutics for Digital Mental Health. JAMA Psychiatry. 2019;76:1223. 10.1001/jamapsychiatry.2019.2075. 31433448 10.1001/jamapsychiatry.2019.2075 PMC7271442

[28] Graham AK, Wildes JE, Reddy M, Munson SA, Barr Taylor C, Mohr DC. User-centered design for technology‐enabled services for eating disorders. Intl J Eat Disorders. 2019;52:1095–107. 10.1002/eat.23130. 10.1002/eat.23130 PMC7265747 31313370

[29] McCurdie T, Taneva S, Casselman M, Yeung M, McDaniel C, Ho W, et al. *mHealth Consumer Apps*: The Case for User-Centered Design. Biomedical Instrum Technol. 2012;46:49–56. 10.2345/0899-8205-46.s2.49. 10.2345/0899-8205-46.s2.49 23039777

[30] Kujala S. User involvement: A review of the benefits and challenges. Behav Inform Technol. 2003;22:1–16. 10.1080/01449290301782.

[31] Kushniruk A, Nøhr C. Participatory Design, User Involvement and Health IT Evaluation. Stud Health Technol Inf. 2016;222:139–51. 27198099

[32] Macchini M, Chiaravalli M, Zanon S, Peretti U, Mazza E, Gianni L, et al. Chemotherapy in elderly patients with pancreatic cancer: Efficacy, feasibility and future perspectives. Cancer Treat Rev. 2019;72:1–6. 10.1016/j.ctrv.2018.10.013. 30414985 10.1016/j.ctrv.2018.10.013

[33] Corsini MM, Miller RC, Haddock MG, Donohue JH, Farnell MB, Nagorney DM, et al. Adjuvant Radiotherapy and Chemotherapy for Pancreatic Carcinoma: The Mayo Clinic Experience (1975–2005). JCO. 2008;26:3511–6. 10.1200/JCO.2007.15.8782. 10.1200/JCO.2007.15.8782 18640932

