# Supplementary material for: Age-Based Left-Digit Bias in the Treatment of Pancreatic Adenocarcinoma
Source: J Gastrointest Cancer. 2026 Apr 13;57(1):90. doi: 10.1007/s12029-026-01459-1 (PMC13076376; doi:10.1007/s12029-026-01459-1)
Supplement: Supplementary file 1 — Supplementary Material 1 (DOCX 12.5 KB) [file 12029_2026_1459_MOESM1_ESM.docx]

**Table S1. Distribution of chemotherapy sequence by age decade among patients with stage I–II PDAC in the NCDB, 2004–2020.**

| Age decade | Adjuvant | Neoadjuvant | Neoadjuvant + Adjuvant | No chemo | Other/unknown |
| --- | --- | --- | --- | --- | --- |
| 50s (n=16,891) | 4,419 (26%) | 1,809 (11%) | 1,191 (7%) | 8,406 (50%) | 1,066 (6%) |
| 60s (n=30,727) | 7,625 (25%) | 3,164 (10%) | 1,894 (6%) | 16,383 (53%) | 1,661 (5%) |
| 70s (n=30,933) | 6,000 (19%) | 2,248 (7%) | 1,161 (4%) | 19,447 (63%) | 2,077 (7%) |
| 80s (n=18,550) | 1,363 (7%) | 374 (2%) | 148 (1%) | 15,324 (83%) | 1,341 (7%) |
| 90s (n=2,983) | 17 (1%) | 5 (0%) | 1 (0%) | 2,812 (94%) | 148 (5%) |
